# Tracking rivalry with neural rhythms: multivariate SSVEPs reveal perception during binocular rivalry

**DOI:** 10.1093/nc/niae028

**Published:** 2024-06-22

**Authors:** Ruben E Laukkonen, Evan Lewis-Healey, Luca Ghigliotti, Nasim Daneshtalab, Jet Lageman, Heleen A Slagter

**Affiliations:** Health, Southern Cross University, Gold Coast Airport, Terminal Dr, Bilinga, Gold Coast, QLD 4225, Australia; Cognitive Psychology, Vrije Universiteit, De Boelelaan 1117, Amsterdam, North Holland 1081 HV, Netherlands; Cognitive Psychology, Vrije Universiteit, De Boelelaan 1117, Amsterdam, North Holland 1081 HV, Netherlands; Psychology, University of Cambridge, Downing Pl, Cambridge CB2 3EB, United Kingdom; Cognitive Psychology, Vrije Universiteit, De Boelelaan 1117, Amsterdam, North Holland 1081 HV, Netherlands; Cognitive Psychology, Vrije Universiteit, De Boelelaan 1117, Amsterdam, North Holland 1081 HV, Netherlands; Cognitive Psychology, Vrije Universiteit, De Boelelaan 1117, Amsterdam, North Holland 1081 HV, Netherlands; Cognitive Psychology, Vrije Universiteit, De Boelelaan 1117, Amsterdam, North Holland 1081 HV, Netherlands

**Keywords:** binocular rivalry, consciousness, no-report, SSVEP, EEG

## Abstract

The contents of awareness can substantially change without any modification to the external world. Such effects are exemplified in binocular rivalry, where a different stimulus is presented to each eye causing instability in perception. This phenomenon has made binocular rivalry a quintessential method for studying consciousness and the necessary neural correlates for awareness. However, to conduct research on binocular rivalry usually requires self-reports of changes in percept, which can produce confounds and exclude states and contexts where self-reports are undesirable or unreliable. Here, we use a novel multivariate spatial filter dubbed ‘Rhythmic Entrainment Source Separation’ to extract steady state visual evoked potentials from electroencephalography data. We show that this method can be used to quantify the perceptual switch-rate of participants during binocular rivalry and therefore may be valuable in experimental contexts where self-reports are methodologically problematic or impossible, particularly as an adjunct. Our analyses also reveal that ‘no-report’ conditions may affect the deployment of attention and thereby neural correlates, another important consideration for consciousness research.

Binocular rivalry (BR; Wheatstone 1838) is a visual perceptual phenomenon that occurs due to the dichoptic presentation of two distinct images. Presenting a distinct image to each eye separately leads to the two stimuli alternately reaching conscious awareness, as well as the potential fusion of both stimuli for short periods of time during the transition period (also known as ‘mixed percepts’). BR is a popular method in the study of consciousness as it provides a direct measure of what enters individual’s conscious awareness, without changing the stimuli (Frith et al. 1999). BR paradigms have therefore been used over the past several decades to identify necessary factors that permit consciousness and their underlying neural mechanisms (Tong et al. 2006, Brascamp et al. 2018). In other works, BR paradigms have been used to investigate the effects of higher-order phenomena such as subjective value (Balcetis et al. 2012, Wilbertz et al. 2014) and emotional processing (Alpers and Pauli 2006, Alpers and Gerdes 2007, Yoon et al. 2009), on access to awareness as indexed by the ‘waxing’ and ‘waning’ of the rivalry stimuli. However, much like other studies of consciousness, BR paradigms have relied on self-report methods to elucidate the phenomenal experience of participants. Besides confounding neural correlates (Brascamp et al. 2015, Tsuchiya et al. 2015, Sandberg et al. 2016), self-reports can of course be problematic for many reasons, such as response biases (Wenger and Rasche 2006), social desirability effects (Fisher 1993), failures of introspection (Johansson et al. 2006, Carruthers 2007), and they can cause dual-task effects by ‘overshadowing’ the primary task (Chin and Schooler 2008).

In the present study, we aim to use a novel ‘multivariate’ method to extract steady state visual evoked potentials (SSVEPs) during BR known as rhythmic entrainment source separation (RESS) (Cohen and Gulbinaite 2017). We then aim to track perceptual switches during BR using the RESS signal in order to achieve a high-fidelity neural measure of changes in perception that can be used to determine changes in the content of conscious awareness without subjective reports. Previous studies have attempted to measure BR using methods other than self-reports, such as multivariate pattern analysis (MVPA; Sandberg et al. 2013, Baker 2017, Wilbertz et al. 2017, 2018), optokinetic nystagmus (OKN; Frässle et al. 2014, Marx and Einhäuser 2015, Fujiwara et al. 2017), pupil dilation (Naber et al. 2011), and SSVEPs (Brown and Norcia 1997, Alpers et al. 2005, Zhang et al. 2011, Jamison et al. 2015). However, each of these methods suffers from limitations.

In the case of MVPA, a classifier is provided with a training set of data (such as electroencephalography (EEG) or functional magnetic resonance imaging (fMRI) data collected during BR), which is then used to decode what stimulus the participant is perceiving at a specific time in a test set of data. The downside of this approach is that MVPA studies often require a large data set to train the classifier and still tend to achieve relatively low classification accuracy (e.g. ∼62%, Baker 2017). For example, Wilbertz et al. (2017) required up to six baseline 180s BR trials for classification training, and decoding accuracy was 59.16%. Although OKN has achieved relatively high accuracy in tracking perception during BR (e.g. ∼88% accuracy; Frässle et al. 2014), this method requires stimuli that move in different directions, such that when one percept dominates in consciousness, fast eye movements mirror this movement. The issue with this method is that it precludes stimuli with higher-order properties, and it can interfere with the state of interest due to its distracting and visually demanding nature (e.g. during meditation, Carter et al. 2005). It can also foreseeably affect the deployment of attention, which is drawn into the ‘sideways’ movements of the sinusoidal gratings, and therefore may affect rivalry itself (Chong et al. 2005, Paffen et al. 2006, Zhang et al. 2011).

Another popular method to track perception without reports is the SSVEP (Brown and Norcia 1997, Alpers et al. 2005, Zhang, et al. 2006; Jamison et al. 2015, Zhang et al. 2011). Here, an individual is presented with a stimulus that is flickering at a particular frequency, which then elicits corresponding neural responses at the same frequency, known as the SSVEP. The amplitude of the SSVEP signal is associated with visual attention and conscious awareness (but see Davidson et al. 2020) and can provide a psychophysiological correlate of which of multiple stimuli flickering at different frequencies is currently in the mind’s eye (Müller et al. 2003, Cosmelli et al. 2004, Jamison et al. 2015, Vissers et al. 2017). It thus can be used in BR paradigms by applying different flicker frequencies to the stimuli and tracking the relative strength of the different frequencies. In other words, the waxing and waning of the SSVEP can be used to index rivalry.

Typically, these SSVEP extraction processes utilize the ‘best-electrode approach’, whereby a power spectrum analysis reveals the electrode that yields the highest power for the response frequency. The time series from this specific electrode is then used in subsequent analyses. To illustrate the technique, we describe two applications of SSVEPs using EEG in BR upon which our method builds (for later adaptations and applications of these methods, see Tononi et al. 1998, Srinivasan et al. 1999, Cosmelli et al. 2004, Kamphuisen et al. 2008, Sutoyo and Srinivasan 2009, Jamison et al. 2015, Katyal et al. 2016, Roy et al. 2017). Notably, no studies that we are aware of have quantified perceptual switch-rate using SSVEPs and analytically compared them to subjective reports. Yet, being able to objectively index the actual switch-rates of the rivalry stimuli would provide an important novel method to measure changes in attention and conscious perception, including in no-report conditions.

Brown and Norcia (1997) induced BR by dichoptically presenting cosine gratings to eight participants. They flickered one of the gratings at a frequency of 5.5 Hz and the other at 6.6 Hz. Results showed that the SSVEPs, triggered by the two stimuli, were anticorrelated, presumably because the amplitude of the perceived response frequency tended to increase when the amplitude of the unperceived response frequency decreased. The anticorrelation largely disappeared when the orientation of the stimuli was changed such that rivalry was no longer present, thereby linking the amplitude of the SSVEP signal to the perceptual dynamics of BR. Another key study investigated the effects of attention on rivalry using SSVEPs. Zhang et al. (2011) presented checkerboard stimuli to each eye, flickering at different frequencies (6.6 Hz for the left eye, 7.5 Hz for the right eye), and manipulated participants’ attention. In the unattended condition, where participants completed a difficult colour-shape conjunction detection task located at the central fixation point of the stimuli, they failed to find the expected counterphase modulation of the SSVEP signal. This was supported by the finding that an anti-correlation between the amplitudes of the two stimuli, indicative of rivalry (cf. Brown and Norcia 1997), was found in the attended condition, but not in the unattended condition. These two studies provide a valuable proof-of-concept that a no-report SSVEP paradigm can be used to track conscious perception, which would not otherwise be possible.

Brown and Norcia (1997) found that low signal-to-noise ratio (SNR) can be an issue when using SSVEPs within BR, especially over longer time windows. Low SNR reduces the fidelity with which the SSVEP can track changes in percept and may explain why previous research (including Brown and Norcia 1997, Zhang et al. 2011) relied on an overall rivalry ‘index’ based on the anti-correlation of the competing rivalry frequencies. To derive a robust anti-correlation is likely to require many trials, and the highest anti-correlation found by Zhang et al. (2011) was *r* = −0.319, and by Brown and Norcia (1997) ranged from *r* = −0.37 to −0.73 depending on the subject. This measure also does not provide a quantification of the actual switch-rates of the rivalry stimuli, which can be an extremely useful outcome variable when investigating changes to attention and conscious processing in different states (Carter et al. 2005, 2007). Although counterphase modulation of the SSVEP signatures has been demonstrably linked to the conscious awareness of stimuli in rivalry trials (e.g. Lansing 1964, Wang et al. 2004), previous research has either presented single and representative trials illustrating the connection between changes in SSVEP and rivalry along a timecourse and/or averaged SSVEP responses across trials without quantifying switch rates *per se*.

Thus, novel methods are needed to improve the SNR of SSVEP extraction as well as methods that quantify the frequency of perceptual switches over time. The goal of our present study is therefore rather straightforward: we aim to use a spatial filter, known as RESS (Cohen and Gulbinaite 2017), to provide a better index of rivalry switch-rate that might be especially valuable in the study of factors that modulate conscious perception without the possible confounds introduced by self-report, as well as supporting consciousness research in contexts where researchers want to investigate changes to rivalry under non-ordinary states with high-fidelity.

The general idea of RESS is simple: Find a vector that maximally differentiates two covariance matrices—one corresponding to the to-be-maximized feature and one corresponding to the to-be-minimized feature—and then use that vector as weights to combine the data from all electrodes (Cohen and Gulbinaite 2017, pg. 51).

The RESS method has several advantages over traditional SSVEP extraction processes (Cohen and Gulbinaite 2017). For one, it maximizes the potential SNR of specific frequencies through the application of a spatial filter to the raw time series (and suppresses surrounding noise). The spatial filter also allows more data-points to be included in the analysis (e.g. compared to a single ‘best’ electrode), and allows for time series analysis of the time course of the SSVEP. Moreover, it bypasses the issue of finding an electrode/cluster of electrodes, as in the ‘best-electrode approach’, by using a weighted combination of the electrodes to form a single time series (the RESS component, see methods for details). The RESS-based SSVEP is therefore not at the whim of a single electrode or cluster that can be difficult to select, can differ across stimulation frequencies and across individuals, and relies on subjective decision-making. The RESS method has been shown to provide better SNR than single electrode approaches, to be relatively robust to noisy electrodes and artefacts, and to also detect SSVEPs at faster and harmonic flicker frequencies (Cohen and Gulbinaite 2017). Faster flicker rates have the advantage that they are less visible, and hence less disruptive, but the SNR of the SSVEP is lower for higher frequencies (Zhu et al. 2010).

Our primary hypotheses are as follows: we expect that transitions in the power of the RESS-derived SSVEP timeseries (hereafter RESS_rivalry) at the response frequencies will strongly correlate with transitions in perception reported by participants. We also explore whether the RESS_rivalry rate in one block of trials can predict participants’ reported rivalry in another block of trials (consistent with intra-individual stability of BR; Carter and Pettigrew 2003, Van Ee 2005, Patel et al. 2015), thus further evidencing potential as a no-report paradigm. We also aim to test the robustness of the RESS_rivalry method at different frequencies, particularly faster frequencies than used in previous research, and at different trial lengths.

In sum, our study aims to test whether RESS can provide a fruitful new tool for consciousness research to investigate rivalry switch-rates as an objective adjunct to self-reports. Our preregistered hypotheses, analysis plan, and materials can be found at: https://osf.io/aswxn/wiki/home/.

## Methods

### Participants

A power analysis in G*Power (Faul et al. 2009) indicated that we needed 11 participants for an effect size of 0.8 using a one-tailed hypothesis. A large effect size is supported by our pilot data (see https://osf.io/tydnm/) where we found a strong correlation between the turning points of the SSVEPs and the reported perceptual switch-rate (*r* = 0.95) with 13 participants. Based on this information and the sample sizes of previous research with SSVEPs in BR (e.g. Brown and Norcia 1997, Zhang et al. 2011), 16 participants were recruited for the study (*M* age = 21.19, *SD* = 4.12). Ultimately, the proposed paradigm aims to be useable at the single-participant level. The following inclusion/exclusion criteria were applied: fluency in English; between 18 and 30 years of age; no history of epilepsy or relatives with epilepsy, migraine, vision problems, glasses, colour blindness, amblyopia, or diagnosis of psychiatric/neurological conditions. Participants were recruited through the Vrije Universiteit of Amsterdam SONA participation scheme and received research credits or 10 euros per hour for their participation. The study was approved by the ethics committee of the Vrije Universiteit Amsterdam: VCWE-2016-215.

### Design

The study was a 2 (frequency: slow vs fast) × 2 (trial duration: short vs long) repeated measures design. In the ‘slow’ condition, one stimulus flickered at 14 Hz and the other flickered at 17 Hz. In the ‘fast’ condition, one stimulus flickered at 29 Hz and the other flickered at 34 Hz. Faster flicker rates have the advantage that they are less visible, and hence less disruptive, but the SNR of the SSVEP is lower for higher frequencies (Zhu et al. 2010). The trial durations lasted either 60 s (short) or 360 s (long). In each of these conditions, participants reported every time they experienced a ‘switch’ in the percept by pressing a different button for each different image. In addition to the above, participants completed 2 × 360 s trials (slow and fast frequencies) while focusing on reporting mixed percepts. Here, participants pressed one button for ‘mixed’ and another for ‘not mixed’. In a final set of 2 × 360 s trials (slow and fast), participants observed percepts without providing any responses. Participants first practised the task twice (each 120 s), first with slow flickering stimuli (passive viewing), and then while reporting perceptual switches with fast flickering stimuli. These conditions are illustrated in Table 1.

**Table 1.** List of repeated measures binocular rivalry conditions

Time (s)	Frequency (Hz)	Buttonpress	Block
120	Slow: 14/17	None	Practice
120	Fast: 29/34	Switches	Practice
60	Slow: 14/17	Switches	Block switches
60	Fast: 29/34	Switches	Block switches
360	Slow: 14/17	Switches	Block switches
360	Fast: 29/34	Switches	Block switches
360	Slow: 14/17	Mixed	Block mixed
360	Fast: 29/34	Mixed	Block mixed
360	Slow: 14/17	None	Block no-report
360	Fast: 29/34	None	Block no-report

NB: All trial blocks following the practice block were presented in a randomized order.

### Materials

**Figure 1:**
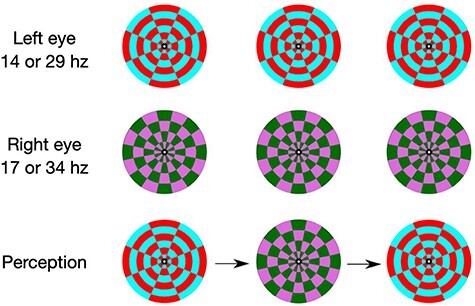
Stimuli presented to participants and corresponding flicker frequencies (NB: 14 Hz was always compared to 17 Hz, and 29 Hz was always compared to 34 Hz).

Stimuli were presented to the participants on a 22-inch Samsung SyncMaster2233 monitor in Psychopy 2 (Peirce et al. 2019) on a black and white background. A red and cyan checkerboard stimulus was presented to the left eye flickering at either 14 Hz or 29 Hz, depending on the trial block, while a green and magenta checkerboard stimulus was presented to the right eye and flickering (colour switching) at either 17 Hz or 34 Hz (see Fig. 1). These frequencies were chosen to avoid the alpha range and to test whether the paradigm was robust to different frequencies (e.g. slower frequencies are known to lead to stronger SSVEPs, Zhu et al. 2010). A mirror stereoscope (Geoscope Standard, Stereo Aids) was used to present the stimuli to each individual eye.

### Procedure

Participants were initially provided with instructions describing the BR task, including examples of the stimuli they would see on a printed handout. This handout included the two different images presented to each eye, as well as a ‘piecemeal’ or ‘mixed’ percept that was a combination of the two. Then, in order to familiarize with the task, participants began with 2 × 120 s practice trials, with both fast and slow flicker frequencies. Adjustments were made as needed to ensure that clear rivalrous perception would occur (e.g. changing spatial coordinates of the stimulus or adjusting the angle of the mirrors in the stereoscope). In the main experiment, participants underwent eight separate BR trial blocks in a randomized order (see Table 1). In four trial blocks labelled ‘block switches’, participants reported when they perceived either stimulus. That is, the participant pressed key ‘1’ if they were primarily perceiving the image presented to the left eye and key ‘3’ if they were perceiving the stimulus presented to the right eye (see Fig. 1). In ‘block mixed’, participants reported mixed percepts by pressing ‘1’ and stable percepts by pressing ‘3’. The reason for separately reporting mixed percepts is to reduce the task demands of having to report all three kinds of percepts at once when stimuli change frequently. Finally, for the no-report trials encompassing ‘block no-report’, participants simply observed the stimuli passively.

### EEG data acquisition and preprocessing

EEG data were collected via 64 electrodes, placed on the scalp using the 10/20 BioSemi ActiveTwo system, with a sampling rate of 512 Hz. EEGLAB in MATLAB (Delorme and Makeig 2004) was used to preprocess the data. The data were re-referenced using the average of two electrodes placed on the participants’ earlobes. The data were high-pass filtered at 0.1 Hz. Excessively noisy channels (Kurtosis Z-score threshold of 8) were rejected and interpolated using the spherical method. No other preprocessing was necessary to remove artefacts such as blinks, as the RESS method suppresses artefacts (Cohen and Gulbinaite 2017).

### Analysis

#### Rhythmic entrainment source separation

The RESS (Cohen and Gulbinaite 2017) method was used to extract the SSVEP signal from the EEG data using an adapted MATLAB script from the authors (see mikecohen.com/data). The RESS method is applied through the construction of linear spatial filters that are used to multiply the time series data collected in the trial blocks, which then creates a separate RESS component time series. The main advantages of the RESS method are 2-fold. First, it maximizes the signal at the frequency of interest (i.e. the response frequency), whilst suppressing the noise. Second, it uses a weighted combination of all electrodes, which allows the RESS component to contain more signal at the designated response frequency. The alternative to this is a univariate approach, where one uses a (single) electrode timeseries with the highest SNR at the frequency of interest. However, in our previous pilot study, we found that the RESS component timeseries outperformed the best-electrode timeseries both in SNR and in associations between rivalry switch rate and turning points of the corresponding timeseries. The RESS method also consistently outperformed the best-electrode approach in SNR in the work by Cohen and Gulbinaite (2017). This is due to the fact that the RESS methodology (i.e. generalized eigendecomposition) has no anatomical constraints in its signal output, meaning we have a multivariate approach to maximize the SNR of the EEG data. Another justification for using generalized eigendecomposition in combination with ‘signal’ (e.g. 14 Hz) and ‘reference’ (e.g. 14 Hz ± 2 Hz) covariance matrices is that we are ‘guiding’ the specific component that we wish to extract from the data. This therefore further allows us to generate the highest SNR at the SSVEP frequency, which is most informative for our analysis purposes. Alternatively, if we were to use a method such as principal components analysis, we would not have as much ‘control’ as to what features we aim to extract from the data, thus making it less useful for our specific analyses.

The general data analysis procedure was as follows: the EEG data were temporally filtered at the response frequency as well as two reference frequencies (above and below the response frequency). This was done by applying a Fourier transform to the EEG signal and multiplying this by a Gaussian distribution. We used a distribution with a full width at half maximum (FWHM) of 0.5 for the response frequency, as this produced the maximum SNR in comparison to other FWHM parameters (cf. Vissers et al. 2017). The neighbouring frequencies (i.e. reference frequencies) were ± 2 Hz away from the response frequency, with a FWHM of 2. An inverse Fourier transform was then applied to the temporally filtered data, allowing analysis in the time-domain. Covariance matrices were then calculated with the response frequency and the neighbouring frequencies. These covariance matrices are used to compute generalized eigendecomposition. Generalized eigendecomposition is used to find the eigenvector with the largest eigenvalue that maximally differentiates the response frequency and the reference frequencies.

In the original paper (Cohen and Gulbinaite 2017), the eigenvalues are used as spatial filters by multiplying the electrode time series with the eigenvalues to create the RESS component. In addition to this, we multiplied the eigenvalues to the temporally filtered data at the response frequency, and applied this whole process to each stimulus frequency, resulting in multiple RESS time series. This is because BR trials using SSVEPs contain information about both response frequencies presented to participants in that trial. Therefore, although the RESS time series suppresses noise and maximizes the signal at the response frequency, signal for the opposing stimulus frequency would still be contained in the original RESS time series. To gain information about the power of the response frequencies in the time-domain, the Filter-Hilbert method was used. The Filter-Hilbert method requires the time series data to be bandpass filtered. Therefore, the bandpass filtered data (i.e. of the response frequency point-wise multiplied by a Gaussian distribution of FWHM = 0.5) were multiplied by the eigenvalues. Here, we applied a Hilbert transform to the bandpass-filtered time series data and computed the squared absolute values of this Hilbert transform. The subsequent generated time series (from now on referred to as the RESS power time series) was computed for both flicker frequencies found in each trial block. The SNR spectrum and topographical plots were also computed for each RESS component.

#### Quantifying perceptual switch-rate

In order to quantify perceptual switches using the RESS method, we used the ‘turning points’ in the power of the RESS-SSVEP timeseries. To identify the turning points, we first used a moving slope function to calculate the difference between the gradients of each time series over a five-data-point window. The moving slope function was used with this five-point window for robustness against noisy data sequences, so that the points of spurious inflection within the time series are less likely to be included. This created two vectors of gradients (one for each RESS power time series). It was then calculated how many successive data points in these vectors went from positive to negative or vice-versa, thus signifying a ‘turning point’ in the time series, creating four variables for each trial block. The mean of these four variables was then computed for each trial block, which could subsequently be compared to self-reported percept switches.

#### Exclusion criteria

During the extraction of the SSVEP, we included certain exclusion criteria to remove excessively noisy trial blocks. The RESS filter is sensitive enough to extract endogenous activity at most frequencies; therefore, in order to ensure that we are extracting the SSVEP signal of interest, we needed to observe localization of the extracted frequency (broadly) to occipital electrodes. Trial blocks where the SSVEP spatial filter was not primarily localized to electrodes along the occipital region were thus removed (i.e. to avoid overfitting or fitting to responses not elicited by the visual SSVEP stimuli, cf. Vissers et al. 2017), which can be caused by movement artefacts, inattention, excessive blinking, or other factors causing inconsistencies in the spatial distribution of the RESS signal (Zhang et al. 2011). In total, we removed 24 trial blocks from fast (29/34 Hz) and 8 from slow (14/17 Hz), and one participant was removed due to violations on all trial blocks. Trial blocks with noisy and excessive gradient changes in the timeseries (>2.5*SD* = 9 trial blocks) were also removed. The first and last 2 s of the trial blocks were excluded due to excessive inflections in the timeseries at the beginning and end of trial blocks. For the final analyses, 49/90 trials from 15/16 participants were included in the analyses, which is a notably higher participant inclusion rate than similar other studies (e.g. Zhang et al. 2011, 4/17 removed; Jamison et al. 2015, 8/23 removed; Srinivasan et al. 1999; 3/11 removed). Most of the trials removed were from the fast flicker frequency condition.

#### Statistical analyses

To examine potential differences in SNR between the flicker frequencies, we first conducted a repeated measures ANOVA with SNR as the dependent measure. SNR was computed separately for each trial block as the average power at the frequency of interest relative to the average power at neighbouring frequency bands (width of 2 Hz, skipping the 0.5 Hz closest to the frequency of interest, i.e. −2.5 Hz to −0.5 Hz relative to frequency of interest and 0.5 Hz to 2.5 Hz relative to frequency of interest). Thus, the SNR is a measure of how strong the frequency of interest is compared to neighbouring frequencies. This is in line with previous studies using SSVEPs (e.g. Zhang et al. 2011; Jamison et al. 2015; Lyu et al. 2020). For further information, please see the original RESS paper (Cohen and Gulbinaite 2017). Next, to determine whether RESS_rivalry can provide an objective measure of perceptual switch rate, we conducted Pearson’s correlations between self-reported perceptual changes and neurally derived changes in percept (confirmatory). These analyses were followed up using multi-level models to ensure that our correlations were robust at both the participant and trial levels (exploratory). We then also ran Pearson’s correlation separately for low- and high-frequency conditions, and for 1- and 6-minute trial blocks, to establish the validity of our measure for different flicker frequencies and to explore the effects of time on rivalry reconstruction from SSVEP signals, respectively (confirmatory). We then aimed to establish whether neurally derived switch-rates in the no-report condition would correlate with participants’ average ‘trait’ switch-rate (confirmatory). A ‘trait’ switch-rate was established by calculating a mean switch-rate using all trials where participants self-reported their perceptual changes. This generalization analysis was followed up with several exploratory analyses (described in the appropriate section) to investigate potential effects of attention and generalization across other conditions. Where appropriate, we also supported our above analyses using Bayesian statistics (e.g. most commonly we report Bayes factor *B*$ + 0$, quantifying a one-tailed Bayesian correlation).

## Results

### SSVEP extraction and timeseries examples

**Figure 2:**
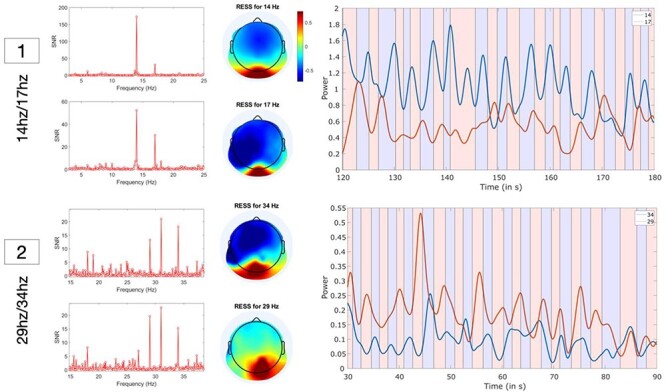
The top images [1] illustrate the SNR, topographical localization, and RESS timeseries for the slower frequencies (14 Hz/17 Hz) for 60 s of one trial block. The bottom images [2] provide the same illustration, but for the faster frequencies (29 Hz/34 Hz). The blue and pink rectangles in the 60 s timeseries plot indicate the participants’ subjective reports [e.g. whether they saw the left (14 Hz; blue) or right (17 Hz; pink) image] and the blue and pink lines represent changes in power of the RESS SSVEP evoked by the two flickering images over time. The average changes in gradient from positive to negative in the RESS power timeseries were used to estimate perceptual switch-rate

**Figure 3:**
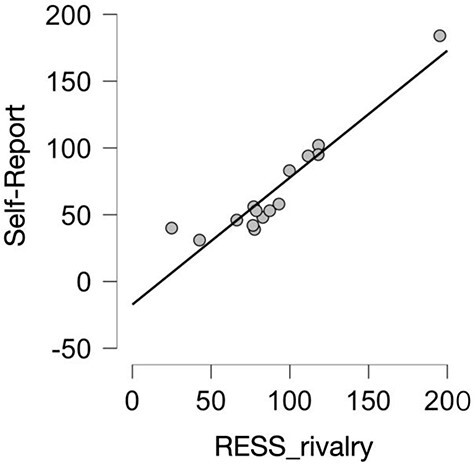
The correlation between participants’ button presses (i.e. average self-reported perceptual switches per trial) and the RESS_rivalry measure of SSVEP based on gradient changes in the timeseries, CI 95% [0.81, 0.99]. Grey dots represent individual participants

Overall, the SSVEP timeseries produced using RESS seemed to provide a reliable measure of perceptual switch-rate during BR. Two representative examples are provided in Fig. 2. In most cases, the RESS signal was strongest at occipital electrode sites (where we expected to find the strongest SSVEP given our visual stimuli) with reasonable to high SNR. Moreover, self-reported switches in perception were closely tied to gradient changes in the RESS timeseries (Fig. 3). We first examined potential differences in SNR between the flicker frequencies. A repeated measures ANOVA revealed a main effect of frequency [*F*(3, 45) = 11.4, *P <* .001]. Tukey comparisons indicated that 14 Hz (*M* = 81.9) provided higher average SNR than 17 Hz [*M* = 21.3; *t*(45) = 4.83, *P* < .001], 29 Hz [*M* = 25.9; *t*(45) = 4.47, *P* < .001], and 34 Hz [*M* = 19.2; *t*(45) = 5, *P* < .001]. No other comparisons were significant. The better SNR for the slower frequency is consistent with previous work that has focused on slower frequencies to elicit the strongest SSVEP signal (Brown and Norcia 1997, Zhang et al. 2011), and explains why more trial blocks had to be removed in these faster frequency conditions.

### RESS compared to self-reports

As described above, we calculated the average perceptual switch-rate (i.e. RESS_rivalry) for each trial block separately by first summating the total number of gradient changes ($gc$) during the trial block for both presented frequencies and then dividing this number by four: $\frac{{{\mathrm{freq}}1\left( {g{c^ + } + g{c^ - }} \right){\ } + {\mathrm{ freq}}2\left( {g{c^ + } + g{c^ - }} \right)}}{4}$. For the first analysis, we generated an average RESS_rivalry for each participant based on the four trial blocks where they reported switches in percept. We found a very strong correlation between self-reported percept changes and the RESS_rivalry measure [*r*(13) = 0.94, *P <*.001] (see Fig. 3). This was supported by a Bayes factor of (*B*$ + 0$) = 102 808, indicating extremely strong evidence for this positive relationship.

In order to check that the correlation between RESS_rivalry and self-reports was robust at both the participant and trial level, we also conducted a multi-level model with participants as the random intercept, SSVEP as the fixed factor, and self-reports as the dependent variable. Here, we also found a strong positive correspondence between self-reports and the RESS_rivalry measure, *b* = 0.88, *t*(41)= 7.91, *P* < .001, CI 95% [0.66, 1.11]. Interestingly, these correlations approximate the within-participant correlation between self-reported trials [short vs long, *r*(31) = 0.84, *P* <.001].

**Figure 4:**
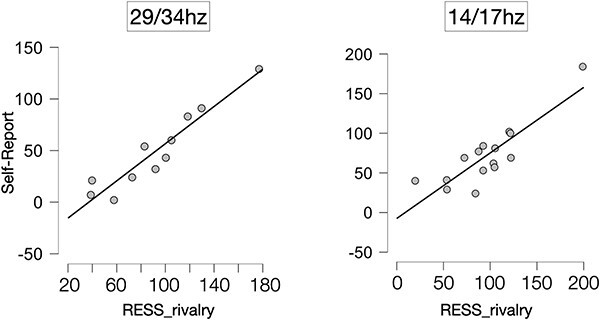
The correlation between participants’ button presses (i.e. average self-reported perceptual switches per trial) and the RESS_rivalry measure of SSVEP based on gradient changes in the timeseries, 29/34 Hz (left) and 14/17 Hz (right). Grey dots indicate individual participants

We then analysed the correspondence between subjective reports and the RESS_rivalry measure for the slow and fast frequencies separately. We found strong correlations between RESS_rivalry for the 29/34 Hz condition, *r*(13) = 0.95, *P* < .001, *B*$ + 0$ = 3387, as well as the 14/17 Hz condition, *r*(13) = 0.86, *P* < .001, *B*$ + 0$ = 1214 (see Fig. 4). The faster flicker frequencies, although providing lower power and SNR than the slower frequencies on average, thus nevertheless provided a very good signal for tracking subjective reports.

We then analysed the 1-minute and 6-minute trial blocks separately in order to explore the effects of time on rivalry reconstruction from SSVEP signals. For 6-minute trial blocks, we found a marginally significant correlation between RESS_rivalry and self-report, *r*(11) = 0.52, *P* = .071, *B*$ + 0$ = 2.860 and the same for 1-minute trial blocks of *r*(12) = 0.19, *P* = .052, *B*$ + 0$ = 0.578. These data may indicate that longer trials provide a better chance of establishing a metric of a participants switch rate using the neurally derived RESS_rivalry; however, these trials were also relatively underpowered. Note that we do not here explore the mixed-report trials using RESS because our method only quantifies switch-rate.

#### No-report and within-subject generalization

**Figure 5:**
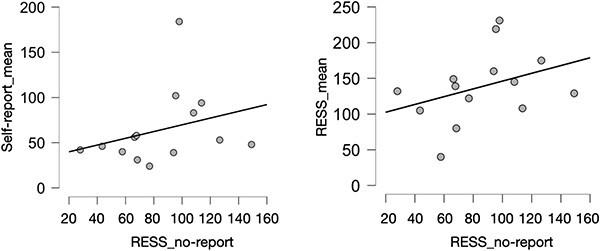
Left: The correlation between participants’ ‘trait’ self-reported switch-rate (average per trial) and RESS switch-rate in the no-report condition. Right: The correlation between participants’ ‘trait’ RESS_rivalry in self-report conditions, and the RESS_rivalry switch-rate in the no-report condition. Grey dots indicate individual participants

We predicted that participants’ average perceptual switch-rate in the self-report conditions (i.e. ‘trait’ switch-rate) would be correlated with RESS_rivalry in the no-report condition. That is, we used participant’s actual perceptual reports as a measure of ‘true perception’ that we tried to predict using SSVEP switch-rates in the no-report condition. This generalization is to be expected if within-participant perceptual rivalry is relatively stable, which is supported by previous works (Carter and Pettigrew 2003, van Ee et al. 2005, Patel et al. 2015). For this analysis, participant five was removed due to an average RESS_rivalry rate >2.5SD above the mean in the no-report condition (496.38 switches), leaving 14 participants for analysis. We found that the switch-rate from the self-report conditions correlated positively with RESS_rivalry in the no-report condition, but this correlation was not significant, *r*(12) = 0.3, *P *= .3, ns, *B*$0 + $ = 1.112, slightly in favour of the null hypothesis (Fig. 5, left). In an exploratory analysis, we tested whether the participants’ average (neurally derived) RESS_rivalry in the self-report conditions was related to RESS_rivalry in the no-report condition, *r*(12) = 0.36, *P *= .1, ns, *B*$ + 0$ = 1.21, slightly in favour of the positive correlation (Fig. 5, right).

#### Does the no-report condition compromise attention?

**Figure 6:**
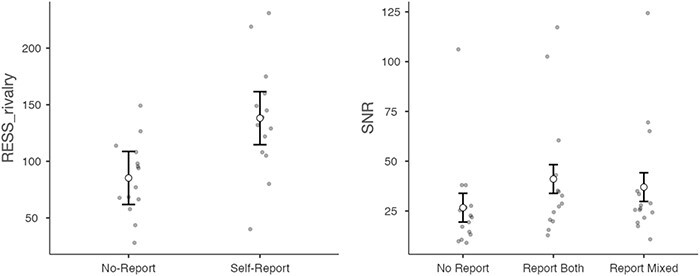
Left: RESS neurally derived switch-rate for no-report versus self-report conditions. Right: SNR ratio in the no-report condition, the self-reported switches condition, and the condition where participants reported mixed percepts. Grey dots indicate individual participants

One possibility for the (relatively) weak correlation between the no-report condition and the other conditions—besides the possibility of being underpowered—is that switch rate is affected by task context (specifically, attention). That is, it is possible that due to the fact that participants do not report their percepts in the no-report condition, they paid less attention to the percepts, which is known to affect rivalry (Ooi and He 1999, Mitchell et al. 2004, Chong et al. 2005, Chong and Blake 2006, Paffen et al. 2006, Hancock and Andrews 2007, Zhang et al. 2011). The first piece of evidence in favour of this idea is that RESS (neurally derived) switch rates were significantly higher in the self-report conditions (*M* = 138.15) compared to the no-report condition (*M* = 85.29), *t*(13) = 4.03, *P* < .001, *d* = 1.08 (Fig. 6, left). This finding is consistent with previous work showing that attention to the rivalry stimuli speeds up switch-rates (Paffen et al. 2006). Moreover, if participants are paying less attention to the stimulus in the no-report condition, we might also see lower SNR in the no-report condition. A repeated measures ANOVA including the factors no-report, switch-report, and mixed-reports revealed a significant effect of condition, *F*(2,28) = 6.93, *P =* .004 (Fig. 6, right). Tukey planned comparisons revealed that the no-report condition (*M = *26.7) had significantly lower SNR than the switch-report (*M = *41.1) [*t*(28) = 3.61, *P* = .003] and mixed-report conditions (*M = *37) [*t*(28) = 2.59, *P* = .039]. There was no significant difference in SNR between the mixed-report and switch-report conditions. The simplest explanation for these results is that the no-report condition was compromised by inattention, consistent with previous work (Zhang et al. 2011).

If our conclusions above are correct—that inattention in the no-report condition weakened the expected correlation with the other conditions—then we may still be able to see generalization of participants’ switch-rates across conditions where they reported their percepts. To this end, we had two (exploratory) hypotheses: first, we expected that participants’ neurally derived switch-rate in the 1-minute condition would relate to participants’ self-reported switch rates in the 6-minute condition. We found this expected relationship, *r*(10) = 0.72, *P *= .008, *B*$ + 0$ = 15.7, which indicates generalization across conditions where participants were presumably attending to the stimuli. Our second hypothesis was that the neurally derived RESS_rivalry switch-rate in the mixed-report condition would predict participant’s self-reported switch-rate in the other conditions where both percepts were reported, *r*(13) = 0.68, *P *= .003, *B*$ + 0$ = 22.1. This correlation further supports the conclusion that the neurally derived RESS_rivalry measure shows intra-individual stability and generalization, as long as participants are responding to the stimuli (Carter and Pettigrew 2003, Van Ee 2005, Patel et al. 2015).^1^

## Discussion

In the present paper, we sought to test the validity of RESS (Cohen and Gulbinaite 2017) as a method for extracting the neural activity associated with changes in conscious perception during BR. In practice, we ‘flickered’ the two stimuli that participants witness during BR at different frequencies. This flicker led to a corresponding evoked response in the EEG signal at a similar frequency. We then extracted this frequency-specific activity from other neighbouring frequencies using a multivariate spatial filter (i.e. RESS). Changes in power over time in the flicker frequency of each stimulus were expected to map onto changes in conscious perception. That is, when one flicker frequency (e.g. 14 Hz) was showing a relative ‘peak’ in activity while the other was showing a relative ‘trough’ (e.g. 17 Hz), then this was taken to indicate that the stimulus tagged with 14 Hz was currently being perceived. We then estimated perceptual switch-rate in a trial by averaging the peaks and troughs of the two frequencies in the RESS timeseries, and found a near ceiling correlation between the neurally ‘inferred’ perceptual switches (i.e. RESS_rivalry) with self-reported shifts in conscious perception. We also provided examples illustrating how the ‘waxing and waning’ of the RESS signal followed a similar trajectory as participants self-reported changes in conscious perception. These results indicate that RESS based on EEG data may provide a valuable objective adjunct to self-reports for BR that may improve traditional SSVEP approaches (Brown and Norcia 1997, Alpers et al. 2005, Zhang et al. 2011, Jamison et al. 2015).

In the experiment, we also presented trials with stimuli flickering at different frequencies (i.e. slower 14/17 Hz and faster 29/34 Hz). Both response frequencies were fast compared to most previous work, which tends to use flicker frequencies below the alpha range (Brown and Norcia 1997, Zhang et al. 2011, Jamison et al. 2015). Part of our goal was to test whether the RESS method would be robust to faster frequencies, which can provide more perceptual stability to the stimulus (at the cost of SNR). Indeed, we found a weaker SNR when extracting the faster frequencies compared to the slowest frequency (14 Hz). However, when correlating the RESS_rivalry measure with self-reports using the faster frequencies (i.e. 29/34 Hz), we still found a near ceiling relationship. This indicates that RESS is likely to be robust to faster frequencies, with the caveat that more trial blocks had to be excluded compared to the slower frequencies.

In terms of functioning as a no-report BR paradigm, the RESS_rivalry measure also seems promising, with some cautions. In the experiment, we included a ‘no-report’ condition where participants did not need to do any task during BR, but simply passively observe the stimuli. We then expected that the inferred switch-rate using RESS_rivalry in this no-report condition would correlate with reported switch-rates in other conditions, due to the known intra-individual stability of BR (Carter and Pettigrew 2003, Van Ee 2005, Patel et al. 2015). However, we failed to find a significant correlation.^2^ We reasoned that this may be due to participants paying less attention in the no-report condition, which would make rivalry dynamics inconsistent across conditions, also possibly affecting the SNR of the SSVEP (Zhang et al. 2011).

The inattention hypothesis was supported by various follow-up analyses. First, the neurally derived switch rate was significantly higher in the report conditions compared to the no-report condition, which is consistent with the finding that attention speeds BR (Paffen et al. 2006). Second, the no-report condition showed significantly lower SNR than the report conditions. Finally, we used the neurally derived RESS_rivalry measure from a self-report condition to predict rivalry in another condition where participants provided different reports (e.g. we correlated RESS_rivalry when participants reported only mixed percepts with RESS_rivalry in the condition in which they reported switches). In each case, we found strong correlations, supporting the ‘no-report inattention hypothesis’, intra-individual stability, and the potential of RESS_rivalry as a no-report paradigm.

Future work that intends to use the RESS_rivalry under no-report conditions may need to ensure that participants are paying attention to the task, e.g. using reward (Ooi and He 1999, Mitchell et al. 2004, Chong et al. 2005, Chong and Blake 2006, Paffen et al. 2006, Hancock and Andrews 2007, Zhang et al. 2011). Nevertheless, the issue of attention is a limitation of any BR paradigm, including report paradigms, since attention may directly affect the presence of rivalry (Zhang et al. 2011) and its temporal dynamics (Carter et al. 2005, Chong et al. 2005, Paffen et al. 2006). Although we may be able to control for physical actions using a no-report paradigm, there remains the issue of so-called ‘mental actions’ (Metzinger 2017) as confounds in the search for the neural correlates of awareness. That is, even if we control for behaviour in the ordinary sense, we cannot easily control for what the participant thinks and attends to. Future work would also benefit from directly comparing RESS_rivalry with another technique such as pupil dilation (Frässle et al. 2014) to determine strengths and weaknesses of the two methods.

Some limitations are worth mentioning. First, EEG has low spatial resolution, which makes it difficult for us to identify specific neural correlates of consciousness during rivalry, for which fMRI might be a better fit. Second, while our study was preregistered and sufficiently powered for large effect sizes, it would be worthwhile replicating these results with a larger population, especially to confirm any smaller effects (e.g. our exploratory analyses). Third, we had to remove a number of trials due to issues extracting the SSVEP from the occipital electrodes, especially for faster flicker frequencies.^3^ This issue may have been circumvented by having trained participants, including a brace for preventing movements, and possibly a more refined preprocessing pipeline (RESS has been shown to be robust without preprocessing, Cohen and Gulbinaite 2017, but not in the context of rivalry). Finally, our goal in the present paradigm was to provide a method for objectively quantifying perceptual switch-rate in BR, which might then be used as an outcome variable in studies of consciousness, including non-ordinary states, emotional processing (where providing self-reports might overshadow the embodied experience), or even individual differences. We did not aim to predict what the individual is seeing with any temporal precision, though some exemplary trials suggest that this is also possible (see also Appendix A for further details). Overall, however, the RESS measure appears to overestimate switch-rates relative to self-report, though it does so consistently and thus, on average, provides a good estimate of switch-rates. Notably, other measures such as pupil dilation and OKN (Naber et al. 2011) also overestimate switch-rates, suggesting that shorter latency transitions may not be available for introspection.

Other no-report BR paradigms, such as OKN and decoding studies, have been used as continuous measures of perceptual status in rivalry trials. However, as discussed in the introduction, these methods also have substantial limitations. For example, OKN stimuli are sinusoidal gratings that move in opposing directions such that they solely contain low-level features. Thus, it may not be possible to use OKN to investigate top-down effects, such as emotional processing (e.g. elicited by meaningful stimuli with higher-order properties). In contrast, SSVEPs have recently been used to predict the phenomenal experience of complex visual stimuli (de Heering et al. 2019), thereby demonstrating a wider stimulus catalogue that can be used with the SSVEP technique in BR. Second, stimuli used within OKN are distracting. This issue is particularly pertinent when seeking to investigate how altered states of consciousness, such as meditation, may affect perceptual switch rate within BR (Carter et al. 2005, 2007).

### A tool for investigating top-down predictive dynamics

Here, we provide just a few case-studies wherein the RESS_rivalry might advance current theorizing in cognitive neuroscience. There is an ongoing and sometimes fierce debate about whether cognition or higher-order processes can affect perception or lower-order processing (Firestone and Scholl 2016). BR, while itself being a part of this debate (Paffen et al. 2006), has also contributed to the debate in important ways. For example, there are studies showing that rivalry dynamics can be affected by physiological states such as hunger (Weng et al. 2019), and higher order processes such as emotion (Alpers and Pauli 2006, Alpers and Gerdes 2007), reward (Marx and Einhäuser 2015), and even gossip (Anderson et al. 2011). However, in most of these studies, one cannot determine whether actual perception is affected by the higher-order ‘cognitive’ process, or if the participant is simply responding differently. Our paradigm could help resolve this limitation by acting as a no-report paradigm or an objective marker complimenting self-reports.

The view that voluntary higher-order states, such as top-down changes in attention, can affect what is perceived during rivalry is also consistent with the remarkable effects of meditation on rivalry. Carter et al. (2005) reported that long-term Buddhist meditators who applied a highly focused form of meditation to rivalry stimuli reported perceptual stability for up to 5 minutes, and one participant reported a prolonged ‘mixed’ percept. Notably, the major limitation of this study was that many participants could not provide self-reports during the practice and thus had to do so retrospectively so as not to disrupt the depth of meditation. In such an experiment, RESS_rivalry could quantify perceptual switch-rate objectively and accurately without disrupting the state, and thereby make it possible to test whether cognition affects perception, by showing that individuals trained in the deployment of attention can, potentially to a remarkable extent, affect what they perceive (see also Katyal and Goldin 2021; Laukkonen and Slagter 2021; Laukkonen et al. 2023). Such findings could also be revealing about the very mechanisms of BR, e.g. by showing that precision-weighting—as formulated in predictive processing accounts of rivalry (Hohwy et al. 2008)—can modulate the importance of prediction errors that drive eventual rivalrous perception.

## Conclusion

The present study tested a recently developed multivariate spatial filter (Cohen and Gulbinaite 2017) as a method for extracting SSVEPs during BR, and then used changes in the timeseries of the extracted SSVEP as a measure of rivalrous perception. The quantification of rivalry switch-rates using ‘peaks and troughs’ of the timeseries showed a near ceiling correlation with self-reported percepts, as well as generalization across trial blocks. The RESS_rivalry method also appeared to be robust to faster frequencies than used in previous work. Our hope is that the RESS_rivalry measure can be used to investigate the top-down effect of higher-order processes on conscious perception during BR as well as provide an objective adjunct to self-reports where they may be insufficient. Our findings also raise the subtle issue of mental actions such as stimulus-related thinking and attentional deployment as potential confounds for identifying true neural correlates of awareness.

## Footnotes

1 See Appendix A for a discussion of exploratory analyses in response to reviewer feedback, as well as technical limitations relevant for future research using faster flicker frequencies.

2 In our pilot project (see https://osf.io/tydnm/), we did however find a significant correlation between a no-report condition and self-reported switches in another condition, albeit with a smaller sample of non-naïve participants.

3 The higher frequencies may reflect the fact that electromyography (EMG) artefacts also load in that frequency range, which may have diminished the capacity of RESS to extract the frequency elicited by the stimulus instead of EMG artefacts.

## Appendix A Exploratory analyses and technical limitations of faster flicker-frequencies

In response to reviewer feedback, we conducted several additional exploratory analyses that aimed to determine dominance durations and more precise temporal dynamics from the RESS timeseries data. Unfortunately, these efforts did not yield satisfactory or consistent results. Here, we outline those attempts and discuss the technical limitations that prevented us from obtaining more fine-grained temporal information about perceptual alternations from the RESS data in the current study.

First, we attempted to identify the specific timing of perceptual switches by examining the difference wave between the two RESS-extracted frequency power time series. The logic was that peaks or troughs in this difference wave may correspond to time points when one percept becomes dominant over the other. However, the difference wave exhibited rapid fluctuations that did not map onto the timing of self-reported perceptual switches in any obvious way. We also tried to identify time points when the two RESS power traces showed peaks in counterphase modulations, reasoning that these may reflect perceptual alternations. But again, there was no clear correspondence between such counterphase peaks and the self-report data.

These difficulties likely stem from the fact that we used relatively high and more widely separated flicker frequencies compared to most previous SSVEP studies of binocular rivalry. The faster frequency oscillated in the RESS power timeseries at a rate nearly four times that of the slower frequency. These divergent oscillation rates, combined with the lower SNR of SSVEPs at higher frequencies, meant that the two RESS traces did not exhibit any simple temporal relationship that could be used to infer the precise dynamics of perceptual switches.

The narrow temporal filtering we applied during RESS extraction also limits our ability to resolve fine-grained temporal dynamics. While using a narrow-band filter centred on the flicker frequency helps isolate the SSVEP signal from background noise and the influence of the competing frequency, it also smears out the temporal resolution of the resulting power timeseries. Attempts to compensate for this using wider filter settings resulted in prohibitively noisy RESS traces that were dominated by non-SSVEP frequencies.

In summary, while the RESS method, as implemented here, appears well-suited for estimating overall perceptual switch rates from SSVEP data, it does not support the recovery of precise switch timing or individual dominance durations within a trial, at least for the range of flicker frequencies we employed. This outcome suggests that when the primary interest is in temporally resolved rivalry dynamics, lower and more closely spaced flicker frequencies may be preferable, as they are likely to yield RESS timeseries with better temporal precision and more easily interpretable interrelationships. Importantly, this limitation does not undermine the validity or utility of the switch rate metric derived here, which could still prove valuable in scenarios where a covert measure of perceptual stability is desirable. However, the lack of temporal precision is an important constraint to bear in mind and one that should be made explicit to others contemplating using this method.

In the future, a finer-grained analysis of the temporal structure of the RESS timeseries, perhaps using more sophisticated signal processing techniques or machine learning approaches, may yet yield meaningful information about the duration of individual dominance periods. Another potentially fruitful direction may be to combine the RESS approach with other covert measures of rivalry dynamics such as optokinetic nystagmus or pupil size measures, capitalizing on the strengths of each method to arrive at a more complete picture of an individual’s perceptual switch patterns. For now though, we emphasize that the primary value of the RESS-derived switch rate metric as a complementary measure to self-report that can provide a useful index of perceptual stability uncontaminated by the act of reporting.
